# Assessment of Accelerometer-Based Physical Activity During the 2017-2018 California Wildfire Seasons

**DOI:** 10.1001/jamanetworkopen.2020.18116

**Published:** 2020-09-30

**Authors:** David G. Rosenthal, Eric Vittinghoff, Geoffrey H. Tison, Mark J. Pletcher, Jeffrey E. Olgin, Donald J. Grandis, Gregory M. Marcus

**Affiliations:** 1 Division of Cardiology, University of California, San Francisco, San Francisco; 2 Department of Epidemiology and Biostatistics, University of California, San Francisco, San Francisco

## Abstract

This cohort study investigates the accelerometer-based physical activity in Californians residing within 10 miles of a monitoring station that registered at least 1 day with an air quality index score greater than 200 during the 2017 to 2018 wildfire seasons.

## Introduction

The risks of industrial pollutants are well documented,^1^ but few studies have examined the public health consequences of climate change–related events such as wildfires. The health benefits of physical activity and harms of sedentary behavior are well established.^2,3^ Although air pollution has been associated with self-reported reductions in physical activity, such ascertainment may be prone to recall bias.^4,5^ Accelerometer-based trackers have become increasingly used to estimate physical activity and can accurately measure step counts.^6^

## Methods

This cohort study was conducted with approval from the University of California San Francisco’s institutional review board and follows the Strengthening the Reporting of Observational Studies in Epidemiology (STROBE) reporting guideline. Informed consent was obtained from participants electronically.

The Health eHeart Study is a worldwide, internet-based, longitudinal cohort study of English-speaking adults aged 18 years or older. Data were accessed for all consenting Health eHeart participants residing in California with Fitbit accelerometer-enabled access to the daily step count data via a public application programming interface. Those with recorded counts of less than 500 total steps or days with wear times less than 10% were excluded.

The daily air quality index (AQI) was determined by accessing the Air Quality System database and matching information from monitoring stations operated by national, state, and local agencies within 10 miles of each participant’s location of residence throughout 2017 and 2018. AQI data were aggregated into Environmental Protection Agency categories used to advise the public regarding associated risks: (1) AQI less than 100 is considered moderate or good air quality, (2) AQI of 101 to 150 is considered unhealthy for sensitive groups, (3) AQI of 151 to 200 is considered unhealthy, and (4) AQI greater than 200 is considered very unhealthy.

Demographic characteristics and comorbidities were determined using survey instruments. Linear mixed models, taking clustering of repeated measures by individual into account, were constructed to analyze the association of AQI with daily step counts. Potential confounders, including age, race, hypertension, coronary artery disease, congestive heart failure, smoking, alcohol abuse, and income, were included in multivariable models. A 2-tailed α less than .05 was considered statistically significant. Data analyses were performed using Stata statistical software version 15 (StataCorp) from July to October 2019.

## Results

Four hundred fifty-five Californians (266 women [58.4%]; mean [SD] age, 52.1 [14.0] years) with connected accelerometer step trackers lived within 10 miles of an air quality station reporting at least 1 daily AQI greater than 200 during the study period. Daily step counts collected over a median (interquartile range) period of 263 (80-603) days and categorized by daily AQI are shown in Table 1. The mean (SE) daily step count for the lowest AQI quartile was 8342 (5321) compared with 7422 (5101) for the highest quartile. Fewer observations with daily step counts and fewer participants contributing data were observed with increasing pollution levels (Table 1). Analysis using the linear mixed model found a statistically significant reduction in daily step counts with progressively worse air quality, with an 18% (95% CI, 11%-24%; *P* < .001) reduction in daily step count when the AQI exceeded 200 compared with AQIs less than 100. Similar results were observed after multivariable adjustment (Table 2).

**Table 1.** Daily Step Counts Stratified by AQI Quartiles

AQI category	Daily observations with step counts, No. (%)^a^	Participants contributing data, No.^b^	Daily steps, mean (SE) [range], No.
<100	144 987 (97.5)	560	8342 (5321) [500-155 826]
101-150	1810 (1.2)	354	8032 (4919) [501-32 948]
151-200	1717 (1.2)	252	7475 (4904) [501-46 553]
>200	155 (0.1)	135	7422 (5101) [797-27 528]

Abbreviation: AQI, air quality index.

^a^ All individuals resided in an area experiencing days in each listed AQI category during the study period.

^b^ Adherence to daily step counts decreased throughout the study period by 12% per year (95% CI, 11%-13%) using a Poisson model with robust SEs.

**Table 2.** Linear Mixed Model Depicting the Unadjusted and Adjusted Association Between Daily Step Count and AQI Category^a^

AQI category	Unadjusted effect size (95% CI)	*P* value	Adjusted effect size (95% CI)	*P* value
<100	1 [Reference]		1 [Reference]	
101-150	0.97 (0.95-0.99)	.002	0.96 (0.94-0.98)	.007
151-200	0.91 (0.89-0.93)	<.001	0.92 (0.90-0.94)	<.001
>200	0.82 (0.76-0.89)	<.001	0.82 (0.75-0.90)	<.001

Abbreviation: AQI, air quality index.

^a^ The relative effect size of daily steps in each AQI category is compared with the reference (AQI ≤100). Effect sizes were adjusted for age, race, hypertension, coronary artery disease, congestive heart failure, smoking, alcohol abuse, and income. Clustering for repeated individual step measurements was taken into account.

## Discussion

Progressively poor air quality was associated with reduced daily step counts, particularly during days with an AQI greater than 200. Although people with increased sensitivity to air pollution are at increased risk for adverse health effects when the AQI exceeds 100, an AQI greater than 200 is deemed hazardous for all individuals and typically results in public health warnings to remain indoors.

This study has several limitations. Location was determined by home address, but we could not determine the actual participant location on a given day or how the entire population behaved in those areas, leading to possible ecological bias. However, as comparisons were performed within individuals over time, it is unlikely that ecological bias resulted in spurious false-positive results. Physical activity inadequately captured by a step tracker (ie, stationary exercise or activity during step tracker charging) was likely not sufficiently captured.

In conclusion, the findings of this cohort study suggest that progressively worse air quality during the 2017 and 2018 California wildfires was associated with a significant reduction in objectively quantified daily step counts. These results highlight the need to account for both direct effects of pollution as well as indirect changes to behavior (ie, less physical activity) when considering the public health implications of climate change–related events such as wildfires.
